# Anaplastic Pleomorphic Xanthoastrocytoma Presenting with Musical Hallucination

**DOI:** 10.1155/2018/6428492

**Published:** 2018-11-11

**Authors:** Oreoluwa Oladiran, Ifeanyi Nwosu, Steve Obanor, Chinyere Ogbonna-Nwosu, Brian Le

**Affiliations:** ^1^Reading Hospital, Tower Health System, Reading, PA, USA; ^2^Leighton Hospital NHS Trust, Crewe, Cheshire, UK; ^3^Maimonides Medical Center, Brooklyn, NY, USA; ^4^Federal Teaching Hospital Abakaliki, Nigeria

## Abstract

Musical hallucinations are a relatively rare form of auditory hallucination characterized by hearing of music in the absence of any external stimuli. This phenomenon has been linked to both psychiatric and structural lesions. We present the case of a previously healthy young male whose presentation with musical hallucinations led to the diagnosis of a rare tumour, anaplastic pleomorphic xanthoastrocytoma.

## 1. Case Presentation

**Figure 1:**
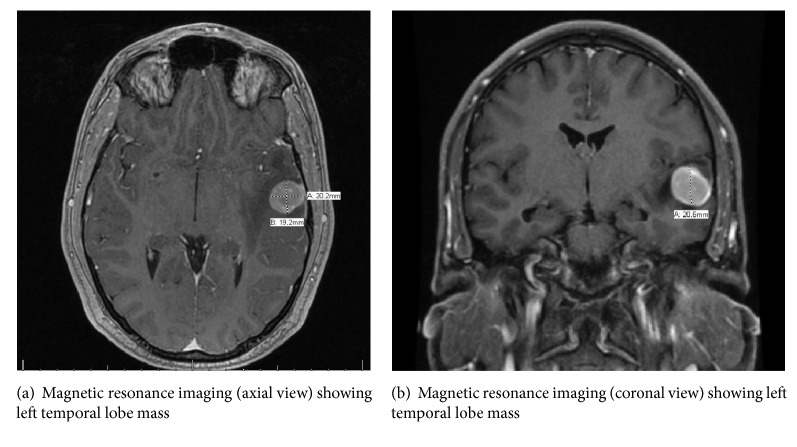


A 28-year-old right hand dominant male with no significant past medical history presents to the emergency department following a motor vehicle accident in which his car was hit from the rear by another vehicle. Patient could not precisely remember the events leading up to the accident, but he thought he might have had a brief episode of loss of consciousness prior to the incident and was awakened when his airbag deployed. He complained of headaches and mild soreness in his neck but denied pain in any other parts of his body. He denied previous seizure, mood changes, or visual disturbances. He does not drink alcohol but smokes tobacco and marijuana occasionally. On further questioning, he reported that over the preceding 2 months, he had been hearing music playing in his ears persistently and loud enough to interfere with his daily activities. He works as an office clerk. Vital signs were normal and physical examination including detailed neurologic examination was otherwise unremarkable. Laboratory tests revealed normal complete blood count and basic metabolic panel; lactic acid was elevated at 6.0 meq/L (normal 0.6-1.4 meq/L). His blood alcohol concentration was <0.01 g/dL (normal <0.01 g/dL) and his urine drug screen was negative for drugs of abuse. Computed tomography (CT) scan of the chest, abdomen, and pelvis was normal. CT head revealed left temporal lobe white matter edema with findings consistent with underlying mass. MRI brain (Figures 1(a) and 1(b)) revealed 2.0 × 1.9 × 2.1 cm homogenous intra-axial neoplasm of the left temporal lobe with reactive vasogenic edema. He was initially commenced on high dose steroids and Levetiracetam for seizure prophylaxis. Following further blood work-up, he gave consent and was taken to the operating room (OR) where left temporal craniotomy for resection of brain mass was performed.

**Figure 2:**
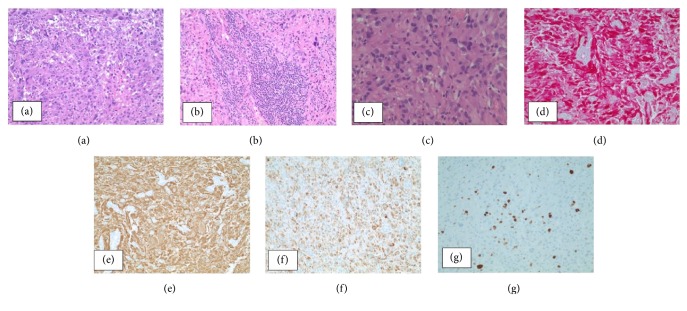
(a) Histologic sections showing a cellular proliferation of pleomorphic cells, some of which are multinucleated (H&E stain, 100x original magnification); lymphocytic infiltration is also seen (b); (c) neoplastic cells show eosinophilic cytoplasm and intracytoplasmic vacuoles, with mitotic figures identified (H&E stain, 400x original magnification); immunohistochemistry shows diffuse reactivity for S-100 (d), GFAP (e), and neurofilament (f); the Ki-67 proliferative index is moderately elevated (g).

Histopathologic examination demonstrates a proliferation of markedly pleomorphic cells, with variation in sizes and shapes. Some cells are multinucleated (Figure 2(a)). Lymphocytic infiltration is focally seen (Figure 2(b)). Neoplastic cells show prominent eosinophilic cytoplasm, with intracytoplasmic vacuoles (Figure 2(c)). Mitotic figures are readily identified, greater than 5 per 10 high-power fields. No evidence of microvascular proliferation or of necrosis is observed. On immunohistochemical assessment, there is diffuse reactivity for S-100 protein (Figure 2(d)) and GFAP (Figure 2(e)), supporting astrocytic origin. Immunoreactivity for neurofilament is also observed (Figure 2(f)). The Ki-67 proliferative index is moderately elevated (Figure 2(g)). The morphologic features, in consideration of immunophenotype, are diagnostic of anaplastic pleomorphic xanthoastrocytoma (WHO grade III). Further molecular testing revealed the presence of a BRAF V600E mutation.

His musical hallucinations resolved postoperatively and given the rarity and likelihood of recurrence of the tumour, he was referred to a tertiary cancer center for further management.

## 2. Discussion

Anaplastic pleomorphic xanthoastrocytoma (APXA), an extremely rare primary brain tumour, is a WHO grade III astrocytoma histologically defined by increased mitotic activity (≥5 mitoses /10 high-power field), with or without necrosis, and clinically notorious for its poor prognostic outcome compared to its benign variant the pleomorphic xanthoastrocytoma (PXA), a low-grade astrocytoma with more favourable prognosis first described by Kepes et al. in 1979 [1, 2].

APXA mostly occur in the first and third decade of life, and similar to PXA they tend to assume a superficial location in the cerebral cortex and leptomeninges with a predilection for the temporal and parietal lobes in majority of the cases [3, 4]. Rarely tumours have been seen in the ventricular system [5, 6].

These tumours mostly originate from malignant transformation of PXA in about 9-20%, but very rarely can arise de novo described as primary APXA [7]. Several cases of primary APXA have been reported in literature [4, 8]. The BRAF-V600E mutation has been implicated in about 78% of PXA and a significant proportion of APXA [9, 10]. The BRAF gene encodes the BRAF protein important in regulating cell growth. V600E is a BRAF gene mutation substituting valine for glutamic acid at position 600 [9]. More than half of the APXA have been found to be V600E-negative, and this is of therapeutic importance as the BRAF inhibitor vemurafenib used in PXA has not shown to be successful in V600E-negative APXA [9]. In this subgroup, various genetic alterations including mutation in the *β*3-*α*C loop of BRFA, copy number alteration, and BRAF fusion have been identified [9]. Isocitrate dehydrogenase (IDH) gene mutation has been described in association with WHO grade II and grade III with various subdivision notably IDH-mutant, IDH-wild type, and the NOS category. IDH-mutant category is seen in 50-70% of anaplastic astrocytoma [11]. IDH mutation may alter the natural history, clinical presentation, and therapeutic response of anaplastic gliomas but its overall prognostic relevance is yet to be fully understood [12, 13].

Most notable findings on histological examination of APXA are pleomorphic, xanthomatous tumour cells with intracytoplasmic lipid vacuoles. Multinucleated giant cells, increased mitotic activity, hyperchromatism, cell atypia, nuclear irregularities, and necrosis may be seen [14]. Absence of palisading necrosis and endothelial microvascular proliferation enables pathologist distinguish these tumours from majority of glioblastomas [15]. APXA can be difficult to differentiate histologically from two rare subtypes of glioblastoma multiforme (GBM), namely, epithelioid GBM (E-GBM) and giant cell GBM (GC GBM). In this scenario, the presence of intracytoplasmic vacuoles along with prominent lymphocytic infiltration and the absence of microvascular proliferation and necrosis favoured a diagnosis of APXA [16].

Typically, most of these patients present with epileptic seizure. Interestingly is the bizarre/atypical pattern of presentation in our patient with musical hallucination, and to the best of our knowledge only one case of glioblastoma multiforme presenting primarily as MH has been reported in literature [17].

Auditory hallucinations are defined as the conscious experience of sounds occurring in the absence of actual sensory input, a minority of which are musical hallucinations where a subject hears music when none is playing as was the case in this patient. Musical hallucination (MH) is a very rare presentation of gliomas. MH a type of “formed” auditory hallucination where patient clearly perceives musical sound, instrumentation, lyrics, and songs [18, 19]. They can be a manifestation of psychiatric as well as organic diseases. MH are manifestations of common psychiatric disorders such as obsessive-compulsive disorders, schizophrenia, and anxiety, but can be seen in organic diseases including sensorineural deafness, brain stem tumours, subarachnoid haemorrhage, and thalamic infarcts [20, 21]. Due to the rarity of this tumour and dearth of literature a standard management guideline has not been established. Standard surgical excision followed by radiotherapy or chemotherapy including new alkylating agents such as temozolomide has not been successful as noted with PXA [14, 22]. Temozolomide and BRAF inhibitor vemurafenib are effective in PXA irrespective of the V600E tumour status [23]. Gross total resection remains the goal of treatment although reports of improved survival have been documented with stereotactic radiosurgery [14, 22]. This article emphasizes the need to always consider organic lesions in the differential diagnosis of patients with auditory hallucinations.

## 3. Conclusion

APXA is a rare entity and more so atypical is its presentation as musical hallucination. A high index of suspicion is therefore needed to exclude structural brain abnormalities in young patients presenting with typical psychiatric symptoms. Total resection remains the standard treatment of this rare tumour as limited benefits have been observed from chemotherapy and radiotherapy.
